# Auto Arginine-GlcNAcylation Is Crucial for Bacterial Pathogens in Regulating Host Cell Death

**DOI:** 10.3389/fcimb.2020.00197

**Published:** 2020-05-05

**Authors:** Juan Xue, Xing Pan, Ting Peng, Meimei Duan, Lijie Du, Xiaohui Zhuang, Xiaobin Cai, Xueying Yi, Yang Fu, Shan Li

**Affiliations:** ^1^Institute of Infection and Immunity, Taihe Hospital, Hubei University of Medicine, Shiyan, China; ^2^College of Life Science and Technology, Huazhong Agricultural University, Wuhan, China; ^3^College of Biomedicine and Health, Huazhong Agricultural University, Wuhan, China; ^4^School of Medicine, Southern University of Science and Technology, Shenzhen, China

**Keywords:** arginine-GlcNAcylation, auto-modification, T3SS effectors, bacterial pathogen, death receptor signaling, NleB, SseK

## Abstract

Many Gram-negative bacterial pathogens utilize the type III secretion system (T3SS) to inject virulence factors, named effectors, into host cells. These T3SS effectors manipulate host cellular signaling pathways to facilitate bacterial pathogenesis. Death receptor signaling plays an important role in eukaryotic cell death pathways. NleB from enteropathogenic *Escherichia coli* (EPEC) and SseK1/3 from *Salmonella enterica* serovar Typhimurium (*S*. Typhimurium) are T3SS effectors. They are defined as a family of arginine GlcNAc transferase to modify a conserved arginine residue in the death domain (DD) of the death receptor TNFR and their corresponding adaptors to hijack death receptor signaling. Here we identified that these enzymes, NleB, SseK1, and SseK3 could catalyze auto-GlcNAcylation. Residues, including Arg13/53/159/293 in NleB, Arg30/158/339 in SseK1, and Arg153/184/305/335 in SseK3 were identified as the auto-GlcNAcylation sites by mass spectrometry. Mutation of the auto-modification sites of NleB, SseK1, and SseK3 abolished or attenuated the capability of enzyme activity toward their death domain targets during infection. Loss of this ability led to the increased susceptibility of the cells to TNF- or TRAIL-induced cell death during bacterial infection. Overall, our study reveals that the auto-GlcNAcylation of NleB, SseK1, and SseK3 is crucial for their biological activity during infection.

## Introduction

Death receptor signaling plays an important role in the innate immune system (Park et al., 2007; Wilson et al., 2009; Giogha et al., 2014; Luo et al., 2015). Hijacking this signaling pathway through type III secretion system (T3SS) effectors has been evolved to be a pathogen evasion strategy (Lu et al., 2015; Luo et al., 2015). Previously, we and other groups have shown that an enteropathogenic *Escherichia coli* (EPEC) T3SS effector, NleB, possessed novel arginine GlcNAc transferase activity toward multiple death domain (DD)-containing proteins in the host, including tumor necrosis factor receptor 1 (TNFR1), TNFR1-associated death domain protein (TRADD), FAS-associated death domain protein (FADD), and receptor-interacting serine/threonine-protein kinase 1 (RIPK1) (Li et al., 2013; Pearson et al., 2013). NleB specifically modifies a conserved arginine in the DD containing-proteins, including Arg235 in TRADD, Arg117 in FADD, and Arg603 in RIPK1. This modification blocks homotypic/heterotypic DD interactions, thereby subverting TNF signaling as well as FAS ligand and TRAIL-induced cell death (Li et al., 2013; Ding et al., 2019). More importantly, the glycosyltransferase activity of NleB is crucial for the colonization ability of A/E bacteria during infection in animal models (Li et al., 2013; Pearson et al., 2013; Scott et al., 2017; Ding et al., 2019).

In fact, arginine-GlcNAcylation is not unique to extracellular bacterial pathogen EPEC and enterohemorrhagic *Escherichia coli* (EHEC) (Li et al., 2013; El Qaidi et al., 2017; Ding et al., 2019). Intracellular pathogen *Salmonella entrica* strains secrete three NleB orthologs named SseK1, SseK2, and SseK3 (Kujat Choy et al., 2004; Brown et al., 2011; Yang et al., 2015; El Qaidi et al., 2017; Gunster et al., 2017; Newson et al., 2019). Notably, several studies have provided evidence that SseK1, SseK2, and SseK3 function as GlcNAcylation transferases as well, both *in vitro* and *in vivo* (El Qaidi et al., 2017; Gunster et al., 2017; Newson et al., 2019). In addition, crystal structure studies have revealed that NleB and SseK1/2/3 belong to the GT-A fold glycosyltransferase family (Esposito et al., 2018; Park et al., 2018; Ding et al., 2019; Newson et al., 2019). However, in the study of SseK1/2/3 activities and host targets yield contradictory conclusions. One recent proteomics study together with our study have indicated the preferential substrate(s) is TRADD for SseK1, TNFR1 for SseK3 during *Salmonella* infection (Newson et al., 2019; Xue et al., 2019).

Two previous studies have revealed that in addition to GlcNAcylation of host DDs, NleB/SseKs could also GlcNAcylate themselves when over-expressed. However, the functional importance of this auto-modification is completely unknown (Park et al., 2018; Newson et al., 2019). Here, we identified the percentage and modification sites of this auto-arginine-GlcNAcylation by mass spectrometry analysis. The auto-modification site mutants abolished or attenuated the capability of enzyme activity toward their death domain targets. Loss of auto-GlcNAcylation of NleB, SseK1, and SseK3 led to the increased susceptibility of the host cells to TNF- or TRAIL-induced cell death during infection. Overall, our work highlights the importance of auto-GlcNAcylation of NleB, SseK1, and SseK3 in their biological activity during infection.

## Materials and Methods

### Bacterial Strains and Plasmids Construction

Bacterial strains and plasmids used in this study were listed in Supplementary Tables S1–S3. DNAs for *nleB* and *sseK1/2/3* genes were inserted into pCS2-EGFP and pCS2-3Flag vectors for mammalian expression, and inserted into pET28a vectors for protein expression in *E. coli*. For complementation in EPEC, DNA for NleB and NleB mutants was ligated into the pTRC99A vector under the trc promoter. For complementation in *S*. Typhimurium strain, DNAs for SesK1, SseK2, and SseK3, together with their upstream promoter regions, were inserted into pET28a vector. Human cDNAs for TRADD, TRADD DD, TNFR1 DD, FADD, and RIPK1 DD were amplified from a HeLa cDNA library as previously described (Li et al., 2013). All point mutations were generated by QuickChange site-directed mutagenesis kit (Stratagene). All plasmids were verified by DNA sequencing.

### Antibodies and Reagents

Antibodies for Arg-GlcNAc (ab195033) and DnaK (8E2/2) were purchased from Abcam. Antibodies for Flag M2 (F2426) and α-tubulin (T5186) were Sigma products. Horse radish peroxidase (HRP)-conjugated goat anti-mouse IgG (NA931V) and HRP-conjugated goat anti-rabbit IgG (NA934) were purchased from GE Healthcare. Cell culture products were from Invitrogen, all other reagents used in this study were Sigma-Aldrich products unless specially noted.

### Cell Culture and Transfection

293T cells and HeLa cells obtained from the American Type Culture Collection (ATCC) were grown in DMEM (GIBCO) medium supplemented with 10% FBS (GIBCO), 2 mM L-glutamine (GIBCO), 100 U/ml penicillin, and 100 mg/ml streptomycin (GIBCO). Vigofect and jetPRIME were used for transient transfection following the respective manufacturer's instructions.

### Immunofluorescence and Immunoprecipitation

Cells were fixed with 4% paraformaldehyde for 10 min at room temperature, then treated with 0.2% Triton X-100 for 15 min. After that, cells were blocked with 2% BSA for 30 min, followed by the incubation with the indicated primary antibody and secondary antibody. All image data shown are representative of at least three randomly selected fields. The immunoprecipitation assay with Flag M2 beads was performed as the manufacturer's instruction.

### Protein Expression and Purification

Recombinant protein expression was induced in *E. coli* BL21 (DE3) strain (Novagen) at 22°C for 16 h with 0.4 mM isopropyl-b-D-thiogalactopyranoside (IPTG) after the absorbance of 600 nm reaching 0.8. Affinity purification of LFn-NleB/SseK1/SseK2/SseK3 and His-NleB/SseK1/SseK2/SseK3 proteins was performed using Ni-NTA agarose (Qiagen) following the manufacturer's instructions.

### Circular Dichroism (CD) Spectra of Proteins Secondary Structure

CD spectra in the “far UV” region (185–260 nm) was used to determine the NleB/NleB RA, SseK1/SseK1 RA, and SseK3/SseK3 RA proteins secondary structure. The CD spectroscopy is Chirascan (Applied photophysics), and the operating conditions for the spectrum were set as: spectral bandwidth is 1 nm, step size is 0.5 nm, pathlength is 1 mm, the sample concentration is 0.22 mg/ml, solvent is 10 mM sodium phosphate pH 7.4, time-per-point is 3 s, spectral scan temperature is 25°C, and total N_2_ flow is 5.0 l/min. The spectra measurement data was analyzed by the program Spectrum Manager 2, and the CD spectrum of NleB/NleB RA, SseK1/SseK1 RA, and SseK3/SseK3 RA, plotted as CD (mdeg) against wavelength (nm) is shown in figures.

### Bacterial Infection of Mammalian Cells and Cell Death Measurement

HeLa cells were seeded at a concentration of 2 × 10^4^ per well in 96-well plates day before infection. For bacterial infection, a single colony in 0.5 ml LB was incubated overnight at 37°C. EPEC strains were then diluted by 1:40 in DMEM supplemented with 1 mM IPTG and cultured in the presence of 5% CO_2_ at 37°C for an additional 4 h. For *S*. Typhimurium SL1344 infection, the bacterial cultures were diluted by 1:33 in LB (without antibiotics) and cultured for an additional 3 h. Infection assays were performed at a multiplicity of infection (MOI) of 200 in the presence of 1 mM IPTG for 2 h for EPEC, or at MOI of 100 for 30 min for *Salmonella*, with a centrifugation at 800 g for 10 min at room temperature to promote infection. At the end time point of infection, cells were washed four times with PBS and the extra bacteria were killed with 200 μg/ml gentamicin for EPEC, or with 100 μg/ml gentamycin for *Salmonella*. One-hour CHX pretreatment was used to sensitize TRAIL and TNF stimulation of cell death. Fifteen hours later, CellTiter-Glo® Luminescent Cell Viability Assay kit (Promega) was used to detect the cell survival.

### Liquid Chromatography-Mass Spectrometry Analysis of Intact Proteins

The recombinant proteins of NleB, SseK1, SseK2, SseK3, and their mutants were loaded onto a C4 capillary column (MAbPacTM RP, 4 μm, 2.1 × 50 mm, Thermo Scientific, USA), and eluted by a Dionex Ultimate 3000 HPLC system with the following solvent gradient: 5–100% B in 10 min (A:0.1% formic acid; B: 0.1% formic acid/80% acetonitrile isopropanol). The eluted proteins were sprayed into a Q Exactive Plus mass spectrometer equipped with a Heated Electrospray Ionization (HESI-II) Probe. The protein charge envelop was averaged across the corresponding protein elution peaks, and de-convoluted into non-charged forms by the Thermo Scientific Protein Deconvolution program.

### Q Exactive Plus Mass Spectrometry Analysis of Tryptic Peptides

To determine the exact auto-GlcNAcylation sites on NleB, SseK1, SseK2, and SseK3, these purified proteins were separated by SDS-PAGE, and subjected to in-gel trypsin digestion. The final peptide samples were analyzed by the Q Exactive Plus mass spectrometer equipped with nanoflow reversed-phase liquid chromatography (EASY nLC 1200, Thermo Scientific). EASY nLC 1200 was fitted with a Thermo Scientific Acclaim Pepmap nano-trap column (C18, 5 μm, 100 Å, 100 μm × 2 cm) and a Thermo Scientific EASY-Spray column (Pepmap RSLC, C18, 2 μm, 100 Å, 50 μm × 15 cm), and run at 300 nl/min with the following mobile phases (A: 0.1% formic acid; B: 80% CH3CN/0.1% formic acid). The liquid chromatography separation was carried out with the following gradient: 0~8% B for 3 min, 8~28% B for 42 min, 28~38% B for 5 min, 38~100% B for 10 min. Eluted peptides were electro sprayed directly into the mass spectrometer for MS and MS/MS analyses in a data-dependent acquisition mode. One full MS scan (m/z 350–1500) was acquired, then immediately the 10 ions with the highest intensity were selected for MS/MS analyses. Dynamic exclusion was set with repeat duration of 24 s and exclusion duration of 12 s.

### Proteomic Data Analyses

The MS raw data were processed by Proteome Discoverer (Thermo Scientific) and searched against NleB/SseKs protein database downloaded from UniProt. N-Acetylhexosamine addition to arginine (arginine-GlcNAcylation) set as the variable modifications. The precursor mass tolerance was set at 10 ppm and the fragment mass tolerance was set at 0.02 Da. Maximum missed cleavage was set at 2. Both peptide and protein assignments were filtered to achieve a false discovery rate (FDR) < 1%.

### Statistical Analysis

Statistical analysis was performed using Student's *T*-test to compare two experimental groups, and one-way analysis of variance (ANOVA) was used to compare the differences between multiple groups. Significant differences are marked with ^*^P < 0.05, ^**^P < 0.01, and ^***^P < 0.001; n.s, not significant. All results are graphed as means ± SD for triplicate samples.

## Results

### Auto-Arginine-GlcNAcylation Is Observed in NleB and SseK

**Figure 1:**
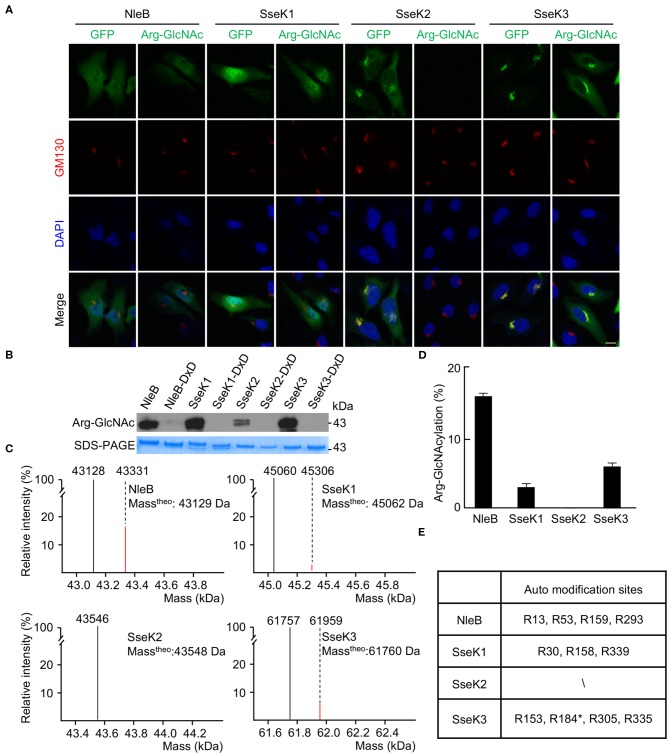
Auto-arginine-GlcNAcylation in NleB and SseKs. **(A)** Subcellular localization of NleB/SseKs effectors. GFP-NleB, GFP-SseK1, GFP-SseK2, and GFP-SseK3 were expressed ectopically in HeLa cells. Shown are immunofluorescence staining of GFP (green), Arg-GlcNAcylated proteins (green), DAPI staining of nuclei (blue) and GM130 staining of the Golgi structure (red) in HeLa cells. Scale bar, 5 μm. **(B)** Analysis of NleB/SseKs' auto arginine-GlcNAcylation by Western blot. Recombinant purified NleB/SseKs and their enzymatic mutants were analyzed on SDS-PAGE gels, followed by immunoblotting with anti-Arg-GlcNAc antibody. **(C)** ESI-MS analysis determination of the total mass of the NleB/SseKs purified from bacteria. The black peak denotes unmodified protein. For NleB and SseK3, the red peak denotes GlcNAcylated form with 203-Da increase, while for SseK1, the red bar denotes GlcNAcylated-acetylated of SseK1 with 245-Da increase. **(D)** Arginine-GlcNAcylation percentage of NleB/SseKs. **(E)** Summary of ESI-MS determination of the modification site of NleB/SseKs. Data in **(A–E)** are representative from at least three repetitions. The asterisk in R184 indicated the auto modification site cited from the Newson's study.

Type III secretion system effectors NleB/SseKs harbor arginine GlcNAc transferase activity and display distinct differences in host substrate specificity (Gao et al., 2013; Li et al., 2013; Pearson et al., 2013; Yang et al., 2015; El Qaidi et al., 2017; Gunster et al., 2017; Scott et al., 2017; Ding et al., 2019; Newson et al., 2019). In order to study the subcellular localization of NleB and SseK proteins, HeLa cells were transfected with the ectopic expression plasmids, pCS2-GFP-NleB and pCS2-GFP-SseK1/2/3. GFP-NleB and GFP-SseK1 were diffusely distributed in the cytoplasm. Accordingly, the arginine GlyNAcylation catalyzed by NleB and SseK1 did not show obvious subcellular localization (Figure 1A). GFP-SseK2 and GFP-SseK3 were found to be co-localized with the Golgi marker GM130. The arginine GlcNAcylation was not detected in GFP-SseK2-transfected cells due to its weak GlcNAc transferase activity. Interestingly, the arginine GlcNAcylation catalyzed by GFP-Ssek3 was co-localized in the host Golgi network (Figure 1A). Considering NleB, SseK1, and SseK3 showed related subcellular localization and modification patterns, we asked if they can modify themselves. To determine whether NleB/SseKs could GlcNAcylate themselves, we purified NleB/SseKs and their dead enzymatic mutants in *E. coli*. These effectors could GlcNAcylate themselves when they were expressed as recombinant proteins in prokaryotic systems (Figures 1B–D). Western blot results showed that NleB, SseK1, and SseK3 were auto-GlcNAcylated, whereas their corresponding dead enzymatic mutants were not. SseK2 was barely able to be auto-GlcNAcylated due to weak enzymatic activity (Figure 1B). Mass spectrometry analysis determined the percentage of 203-Da increase in the total molecular weight of NleB, SseK1, and SseK3 (Figures 1C,D). NleB showed the strongest auto-modification percentage, that was around 15%. SseK2 exhibited little mass change, which was consistent with the weak signal detected by the anti-Arg-GlcNAc antibody (Pan et al., 2014). Recombinant SseK1 and SseK3 showed the auto-modification percentages were 3 and 6%, respectively (Figures 1B–D). Importantly, SseK1 and SseK3 expressing and secreting during *Salmonella* infection showed strong auto-modification. The auto-modification ratio of SseK1 and SseK3 during infection was to the same degree as that of the recombinant purified NleB, which showed a 15% auto-modification ratio (Supplementary Figure 1, Figure 1C). Furthermore, to identify the specific auto-GlcNAcylation sites of NleB and SseKs, we performed the liquid chromatography-tandem mass spectrometry (LC-MS/MS) analysis. A total of 10 arginine residues in three proteins were detected as modification sites. They are Arg13, Arg53, Arg159, and Arg293 in NleB, Arg30, Arg158, and Arg339 in SseK1, as well as Arg153, Arg 305, and Arg335 in SseK3 (Figure 1E, Supplementary Figures 2–4). The modification sites were shown in structures of NleB, SseK1, and SseK3 (Supplementary Figures 5A–C). These findings were similar to a recent report that Arg153, Arg184, Arg305, and Arg335 were the auto-Arg-GlcNAcylation sites of SseK3 in an *in vitro* reconstitution system (Newson et al., 2019).

### Mutation of the Auto-Modification Sites Abolishes the Arginine-GlcNAcylation of NleB, SseK1, and SseK3

**Figure 2:**
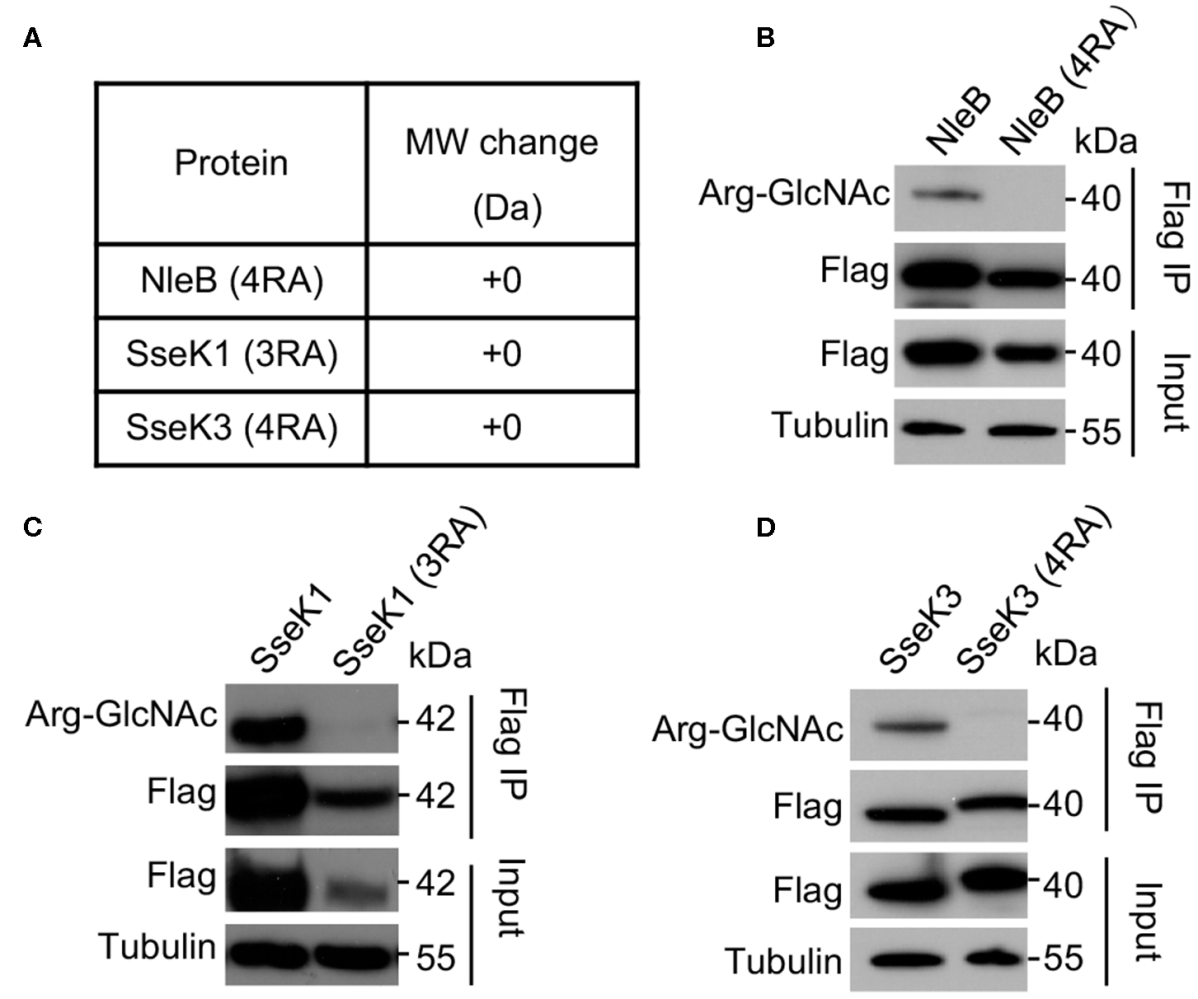
Mutation of the auto modification sites abolishes the arginine-GlcNAcylation of NleB, SseK1, and SseK3. **(A)** Mass spectrometry analysis of the total molecular weight of the indicated auto arginine-GlcNAcylation site-directed mutant proteins. **(B–D)** Effects of the auto Arg-GlcNAcylation of modification site mutation of NleB, SseK1, and SseK3. 293T cells were transfected with the indicated plasmids. Immunoprecipitants (Flag IP) and cell lysates (Input) were loaded onto SDS-PAGE gels, followed by immunoblot with anti-Flag, anti-Arg-GlcNAc, and a loading control anti-tubulin. Data in **(A-D)** are representative from at least three repetitions.

In order to validate the modification sites, mutants of single mutation for each modification site were generated. Single mutations did not abolish the auto-modification. However, mutation of R159 in NleB, R158 in SseK1, and R335 in SseK3 showed decreased auto-modification comparing with their corresponding wild-type proteins and other single residue mutants (Supplementary Figures 5D–F). Then we replaced the arginine residues by alanine residues to generate NleB (4RA) (NleB_Arg13/53/159/293Ala_), SseK1 (3RA) (SseK1_Arg30/158/339Ala_), and SseK3 (4RA) (SseK3_Arg153/184/305/335Ala_) mutants. First, we purified recombinant proteins, NleB (4RA), SseK1 (3RA), and SseK3 (4RA). Mass spectrometry analyses showed that the total molecular weight of these mutants exhibited no 203-Da increase comparing with NleB, SseK1, and SseK3, respectively (Figure 2A). The properties of NleB (4RA), SseK1 (3RA), and SseK3 (4RA) proteins were similar to that of wild type proteins, which were evaluated by ion-exchange, gel-filtration chromatography and circular dichroism (Supplementary Figures 6, 7). Additionally, we verified the auto-GlcNAcylation in a eukaryotic system. 3Flag-NleB, 3Flag-SseK1, 3Flag-SseK3, and their site-directed RA mutants were ectopically expressed in 293T cells. Auto-arginine-GlcNAcylation was measured with anti-Arg-GlcNAc antibody in western blot assay following immunoprecipitation with anti-Flag antibody-conjugated beads. The results showed that the NleB (4RA), SseK1 (3RA), and SseK3 (4RA) mutants completely abolished the auto-arginine-GlcNAcylation signals (Figures 2B–D), indicating that the Arg13/53/159/293 in NleB, Arg30/158/339 in SseK1, and Arg153/184/305/335 in SseK3 were the *bona fide* modification sites.

### Mutation of the Auto-Modification Sites of NleB, SseK1, and SseK3 Abolishes or Attenuates the Enzyme Activity Toward Their Death Domain Targets During Infection

**Figure 3:**
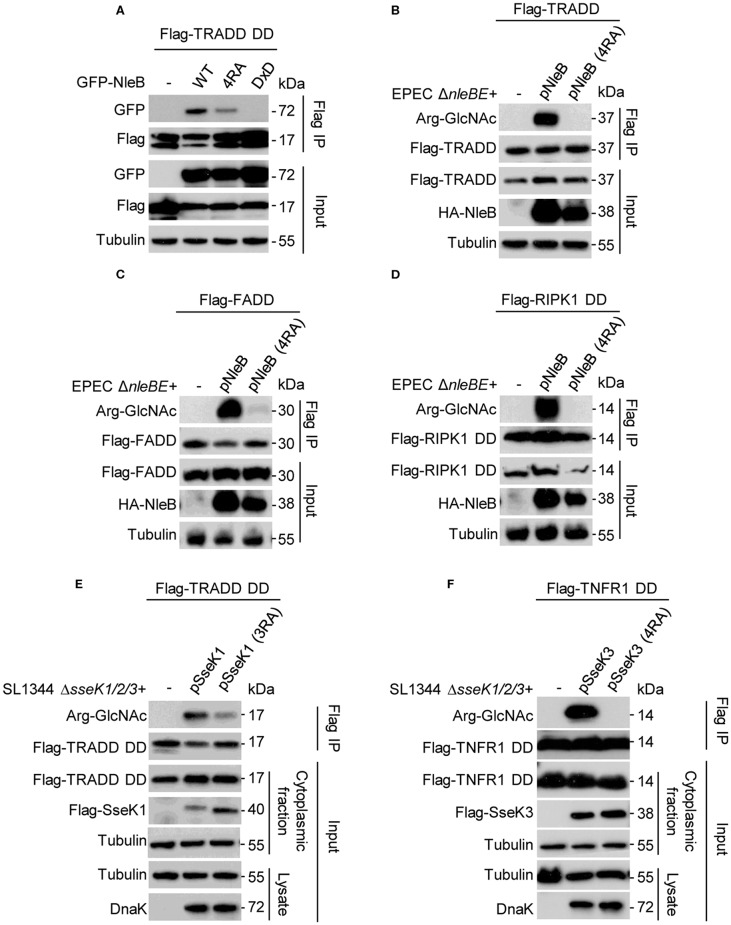
Site-directed mutants of NleB, SseK1, and SseK3 for loss of GlcNAcylation activity toward their DD targets during infection. **(A)** Co-immunoprecipitation of TRADD with NleB or site-directed RA mutant in 293T cells. **(B–F)** Plasmids carrying genes for Flag-TRADD **(B)**, Flag-FADD **(C)**, Flag-RIPK1 DD **(D)**, Flag-TRADD DD **(E)**, and Flag-TNFR1 DD **(F)** transfected into 293T cells were infected with the indicated EPEC or *Salmonella* strains. After infection, cells were lysed and proteins were immunoprecipitated with Flag beads. Samples were analyzed by SDS-PAGE and immunoblotting with indicated antibodies. Data in **(A–F)** are representative from at least three repetitions.

NleB is an inverting glycosyltransferase toward the conserved arginine of TRADD (Arg235), FADD (Arg117), and RIPK1 (Arg603) (Li et al., 2013; Ding et al., 2019). One recent proteomics study have shown that TRADD and TNFR1 are the preferential substrates of SseK1 and SseK3, respectively, during *Salmonella* infection (Newson et al., 2019; Xue et al., 2019). We found that NleB 4RA mutant showed decreased binding ability with TRADD compared to NleB WT (Figure 3A). To verify the biological function of the auto-arginine-GlcNAcylation, we tested whether the auto-modification deficient mutants of NleB, SseK1, and SseK3 could still GlcNAcylate their host targets during infection. Arg-GlcNAc transferase deficient EPEC and *Salmonella* strains were constructed and then expressed with NleB, NleB (4RA), SseK1, SseK1 (3RA), SseK3, or SseK3 (4RA) as indicated (Figures 3B–F). NleB delivered by EPEC could efficiently modify TRADD, FADD, and RIPK1 DD. However, the NleB (4RA) mutant lost the ability to modify these targets, suggesting that the auto-modification sites Arg13/53/159/293 in NleB were essential for the enzymatic activity of NleB (Figures 3B–D). Consistently, SseK1 and SseK3 delivered by *S*. Typhimurium strain could modify TRADD DD and TNFR1 DD, respectively (Figures 3E,F). Whereas, SseK1 (3RA) mutant decreased the enzymatic activity toward TRADD DD, and the SseK3 (4RA) mutant abolished the GlcNAcylation activity toward TNFR1 DD (Figures 3E,F). Further, we evaluated the glycosylation of TRADD DD by NleB single mutants during EPEC infection in order to verify which auto-modification site is important for modification of the host substrate. The NleB mutants R53A and R159A showed decreased glycosylation ability to the host target TRADD DD. Whereas, the mutation of R13A and R293A did not affect the glycosylation on TRADD DD (Supplementary Figure 5G). Taken together, mutation of the auto-modification sites of NleB and SseKs abolished or attenuated their modification on host target proteins. For NleB, the mutation of R53 and R159 severely disrupted its enzymatic activity toward TRADD DD.

### Auto-Arginine-GlcNAcylation of NleB, SseK1, and SseK3 Is Crucial for Their Biological Activity During Infection

**Figure 4:**
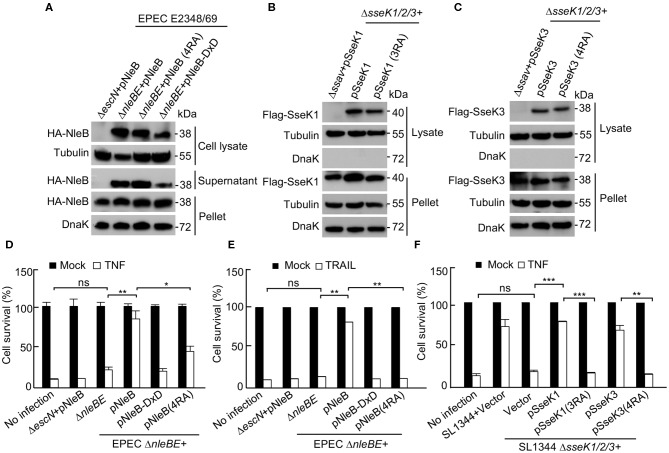
Auto-arginine-GlcNAcylation of NleB and SseK1/3 is crucial for their biological activity during infection**. (A)** Type III-dependent secretion of various NleB mutants. NleB and indicated NleB mutants were expressed in EPEC E2348/69 Δ*nleBE* strain or its type III-deficient mutant strain (Δ*escN*). Secreted and translocated NleB present in the culture supernatant (Supernatant), cell lysate, and total NleB in the bacterial pellet were shown by anti-HA antibody. DnaK and Tubulin were used as a loading control. **(B,C)** Type III-dependent secretion of various SseK1/3 mutants. HeLa cells were infected with the indicated *Salmonella* strains or its type III-deficient mutant strain (Δ*ssav*) for 16 h. Postnuclear extracts were isolated and were analyzed by SDS-PAGE and immunoblotting. Translocated SseK1 **(B)** and SseK3 **(C)** present in the cytoplasmic fraction lysate and total effectors in the bacterial pellet were shown by anti-Flag immunoblots. DnaK and Tubulin were used as a loading control. **(D–F)** Effects of the auto arginine-GlcNAcylation of NleB and SseKs on cell death inhibition. HeLa cells infected with the indicated EPEC and *Salmonella* strains were stimulated with TNF and TRAIL. Cell viability was determined by measuring ATP levels. *P < 0.05, **P < 0.01, ***P < 0.001 by Student's t test, ns, not statistically significant. Means ± SD were shown (*n* = 3). Data in **(A–F)** are representative from at least three repetitions.

SseK1 and SseK3 inhibit cell death during *Salmonella* infection in macrophages (Gunster et al., 2017). The DDs of TRADD, FADD, and RIPK1 can be GlcNAcylated by NleB, resulting in the inhibition of death receptor signaling and nuclear factor-κB (NF-κB) signaling (Li et al., 2013). To assess the biological importance of the auto-arginine-GlcNAcylation, we evaluated the inhibition effect of NleB (4RA), SseK1 (3RA), and SseK3 (4RA) on certain death receptor signaling pathways. First, we determined that the variant forms of NleB (4RA), SseK1 (3RA), and SseK3 (4RA) were translocated into cells at a similar level to that of the WT proteins during infection (Figures 4A–C). Then, HeLa cells were infected with the indicated bacterial strains and stimulated with TNF or TRAIL. NleB delivered by EPEC inhibited both TNF-induced or TRAIL-induced cell death (Figures 4D,E). SseK1 or SseK3 delivered by *Salmonella* was sufficient to inhibit TNF-induced cell death (Figure 4F). However, the auto-modification deficient mutants, NleB (4RA), SseK1 (3RA), and SseK3 (4RA), could not inhibit the death receptor signaling pathways (Figures 4D–F). Therefore, these data suggest that the auto-arginine-GlcNAcylation of NleB, SseK1, and SseK3 is crucial for their biological activity during infection.

## Discussion

Glycosylation is one of the most common post-translational modifications of proteins. Interestingly, more and more evidence indicate a tight junction of this type of post-translational modification with bacterial pathogenicity (Szymanski and Wren, 2005; Ribet and Cossart, 2010; Lu et al., 2015). Besides, T3SS is crucial in many Gram-negative bacteria for injecting effectors, e.g., NleB (Gao et al., 2013; Li et al., 2013; Pearson et al., 2013), SseKs (Kujat Choy et al., 2004; Brown et al., 2011; Yang et al., 2015; El Qaidi et al., 2017; Gunster et al., 2017; Newson et al., 2019), NleE (Nadler et al., 2010; Newton et al., 2010; Zhang et al., 2012), and EspL (Pearson et al., 2017) into host cells, thus manipulating host signaling pathways and evading immune defenses. NleB and SseK1/2/3 behave as unusual arginine GlcNAc transferases, which could modify several different host death domain-containing proteins. In addition to the GlcNAcylation of TRADD, FADD, and RIPK1, two previous studies have noticed that NleB/SseKs could also GlcNAcylate themselves *in vitro* (Park et al., 2018; Newson et al., 2019). However, the physiological significance of the auto-modification remains largely unknown. Here, we firstly report that the auto-GlcNAcylation of NleB, SseK1, and SseK3 is crucial for their biological activity during infection.

We have shown that NleB, SseK1, SseK3, and their related arginine-GlcNAcylation patterns are co-localized, indicating that these T3SS effectors might be auto-GlcNAcylated. Further mass spectrometry analyses identify the auto-modification sites in NleB (Arg13/53/159/293), SseK1 (Arg30/158/339), and Ssek3 [Arg153/184 (Newson et al., 2019) /305/335]. Analyses of SseK3 crystal structure show that R335 locates on the C-terminal lid domain of SseK3, plays a crucial role in UDP-GlcNAc binding, and is also required for the enzymatic activity (Esposito et al., 2018; Park et al., 2018). This might be the reason for Arg335 to be one of the auto-arginine-GlcNAcylation sites for SseK3. Although three or four auto-modification sites are identified in NleB and SseK proteins, 203 Da increase in the total molecular weight indicates only one GlcNAc moiety is added to one molecule. It is intriguing that whether this is a mixture of auto-glycosylated proteins with only one glycosylated residue for each molecule. Meanwhile, we do not know the auto-modification occurs in an intramolecular or intermolecular manner. Thus, the exact molecular mechanism of auto-arginine-GlcNAcylation needs to be further investigated.

Studies have shown that lots of enzymes have been adapted as self-modifying enzymes, and always play a key role in self-activation (Takahashi et al., 2001; Ge et al., 2002; Ding et al., 2003; Uematsu et al., 2007; Black et al., 2008; Blanco-Garcia et al., 2009; Wang and Chen, 2010; Sun et al., 2011; Yuan et al., 2012; Mccullough et al., 2016; Cai et al., 2017). It's well-known that mitogen activated protein kinase kinases (MAPKKs) can activate MAPKs by phosphorylation. Interestingly, in a previous study, Ge et al. reported that p38a was auto-phosphorylated, which acted as another way for its activation (Ge et al., 2002). In addition, Yuan et al. showed that MYST protein was autoacetylated, and the auto-posttranslational-regulation of MYST proteins draws similarities to the phosphoregulation of protein kinases (Yuan et al., 2012).

**Figure 5:**
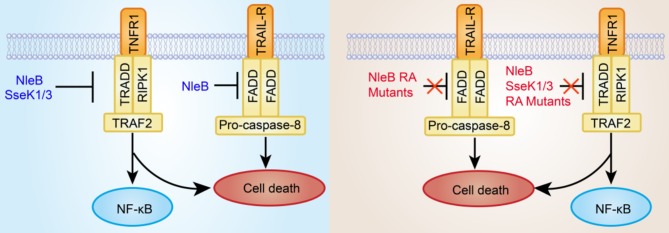
Inhibition of death receptor (TNFR1 and TRAIL-R) signaling by bacterial pathogen T3SS effectors NleB and SseKs or their site-directed RA mutants. The T3SS effector NleB is a glycosyltransferase that catalyzes an unprecedented GlcNAcylation modification on a conserved arginine residue in the death domain of FADD, TRADD, and RIPK1. SseK1/3 is highly homologous to NleB and potentially GlcNAcylates the DD of TRADD and TNFR1, respectively. The modified DD proteins are not recruited to the death receptor complex. Therefore, the cell death is subsequently blocked. However, the site-directed RA mutants of NleB and SseK1/3 almost abolish or attenuate GlcNAcylation activity toward their corresponding host death domain targets, thus failing to inhibit TNF- or TRAIL-induced cell death.

In this study, we show that TRADD, FADD, and RIPK1 are GlcNAcylated by NleB, TRADD is GlcNAcylated by SseK1, and TNFR1 is GlcNAcylated by SseK3 during EPEC or *Salmonella* infection, which are consistent with previous studies (Li et al., 2013; Gunster et al., 2017; Scott et al., 2017; Ding et al., 2019; Newson et al., 2019). In contrast, mutation of the auto-GlcNAcylation sites of NleB, SseK1, and SseK3 abolishes or attenuates their enzymatic activity toward these targets. TRADD is known as the initial adaptor for NF-κB signaling (Hsu et al., 1995; Chen and Goeddel, 2002; Mak and Yeh, 2002; Chen et al., 2008; Pobezinskaya et al., 2008; Pobezinskaya and Liu, 2012; Dowling et al., 2019). RIPK1 and TRADD are synergistically required for TRAIL-induced NF-κB signaling and TNFR1-induced NF-κB signaling and apoptosis (Fullsack et al., 2019). FADD mediates TRAIL-induced necroptosis but antagonizes TNF-induced necroptosis (Kreuz et al., 2004; Grunert et al., 2012; Hartwig et al., 2017; Henry and Martin, 2017; Fullsack et al., 2019). TNFR1, which is the upstream receptor of TRADD, could initiate signaling cascades, including inflammatory cytokine production and programmed cell death, by responding to extracellular TNF (Newson et al., 2019). We and others have found that SseK1 and SseK3 could inhibit TNF-induced cell death during *Salmonella* infection (Gunster et al., 2017; Xue et al., 2019). Compared to wild type NleB, SseK1, and SseK3, loss of auto-GlcNAcylation could not inhibit TNF- or TRAIL-induced cell death. These are consistent with their substrate specificities, indicating that auto-arginine-GlcNAcylation of NleB, SseK1, and SseK3 is crucial for their biological activity during infection (Figure 5).

## Data Availability Statement

The datasets generated for this study are available on request to the corresponding author.

## Author's Note

This article has been released as a Pre-Print at bioRxiv (Xue et al., 2019).

## Author Contributions

SL, JX, and XP conceived the overall study and assisted in the design of experiments. JX and XP conducted and performed the majority of the experiments, analyzed data with the assistance from TP, MD, LD, XZ, XC, XY, and YF. JX, XP, and SL wrote the article. All authors read and approved the final version of the article.

## Conflict of Interest

The authors declare that the research was conducted in the absence of any commercial or financial relationships that could be construed as a potential conflict of interest.
