# Supplementary material for: Auto Arginine-GlcNAcylation Is Crucial for Bacterial Pathogens in Regulating Host Cell Death
Source: Front Cell Infect Microbiol. 2020 May 5;10:197. doi: 10.3389/fcimb.2020.00197 (PMC7214673; doi:10.3389/fcimb.2020.00197)
Supplement: Supplementary file 1 [file Data_Sheet_1.pdf]

Supplementary Material

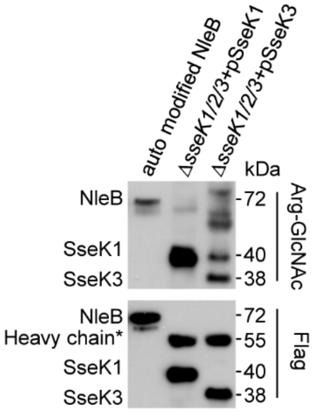

Supplementary Figure 1 Auto-arginine-GlcNAcylation of SseK1 and SseK3 during bacterial infection. HeLa cells were infected with indicated *Salmonella* strains for 16 h. Cells were lysed and proteins were immunoprecipitated with anti-Flag beads. Samples were analyzed by SDS-PAGE and immunoblotted with indicated antibodies. Anti-Flag was shown as a loading control. Auto-modified NleB was used as a reference. The asterisk indicated the heavy chain. Data represent at least three repetitions.

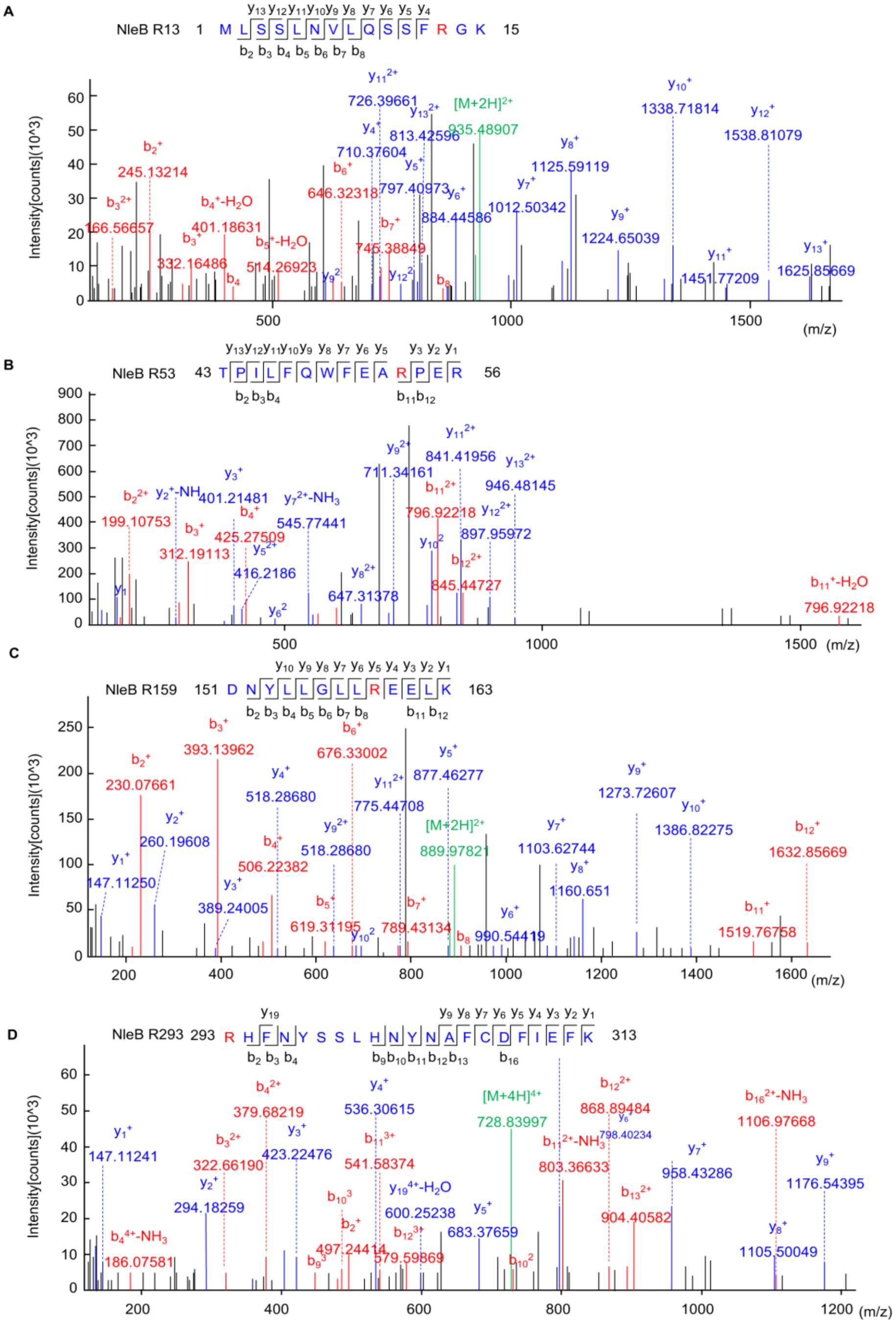

Supplementary Figure 2 HCD analysis of the peptides GlcNAcylated by NleB. HCD mass spectrum of Arg13 **(A)**, Arg53 **(B)**, Arg159 **(C),** and Arg293 **(D)** containing tryptic peptide from NleB in bacteria. The fragmentation patterns of the generated *b* and *y* ions were displayed along the peptide sequence on top of the spectrum.

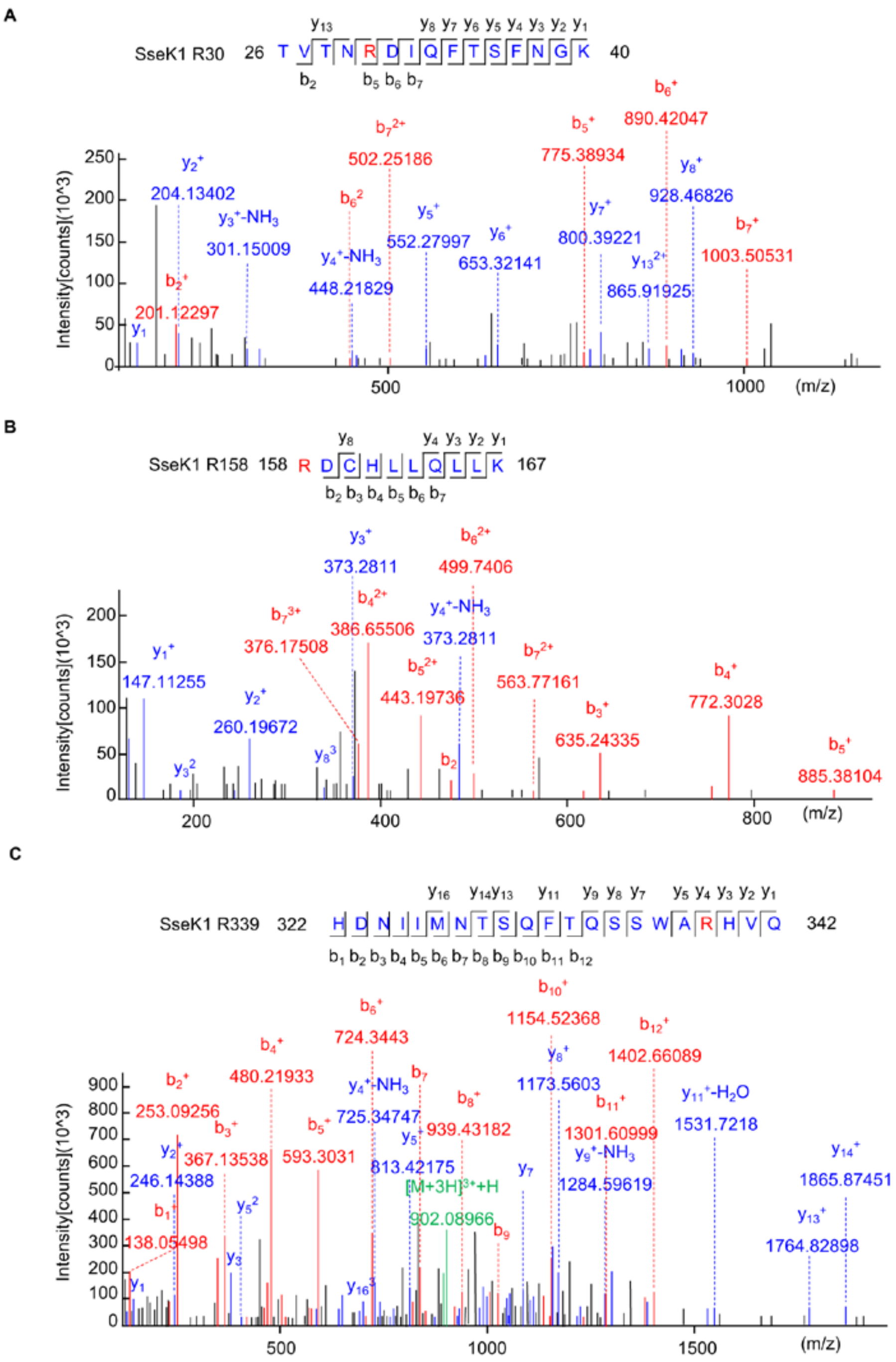

Supplementary Figure 3 HCD analysis of the peptides GlcNAcylated by SseK1. HCD mass spectrum of Arg30 **(A)**, Arg158 **(B)**, and Arg339 **(C)** containing tryptic peptide from SseK1 in bacteria. The fragmentation patterns of the generated *b* and *y* ions were shown along the peptide sequence on top of the spectrum.

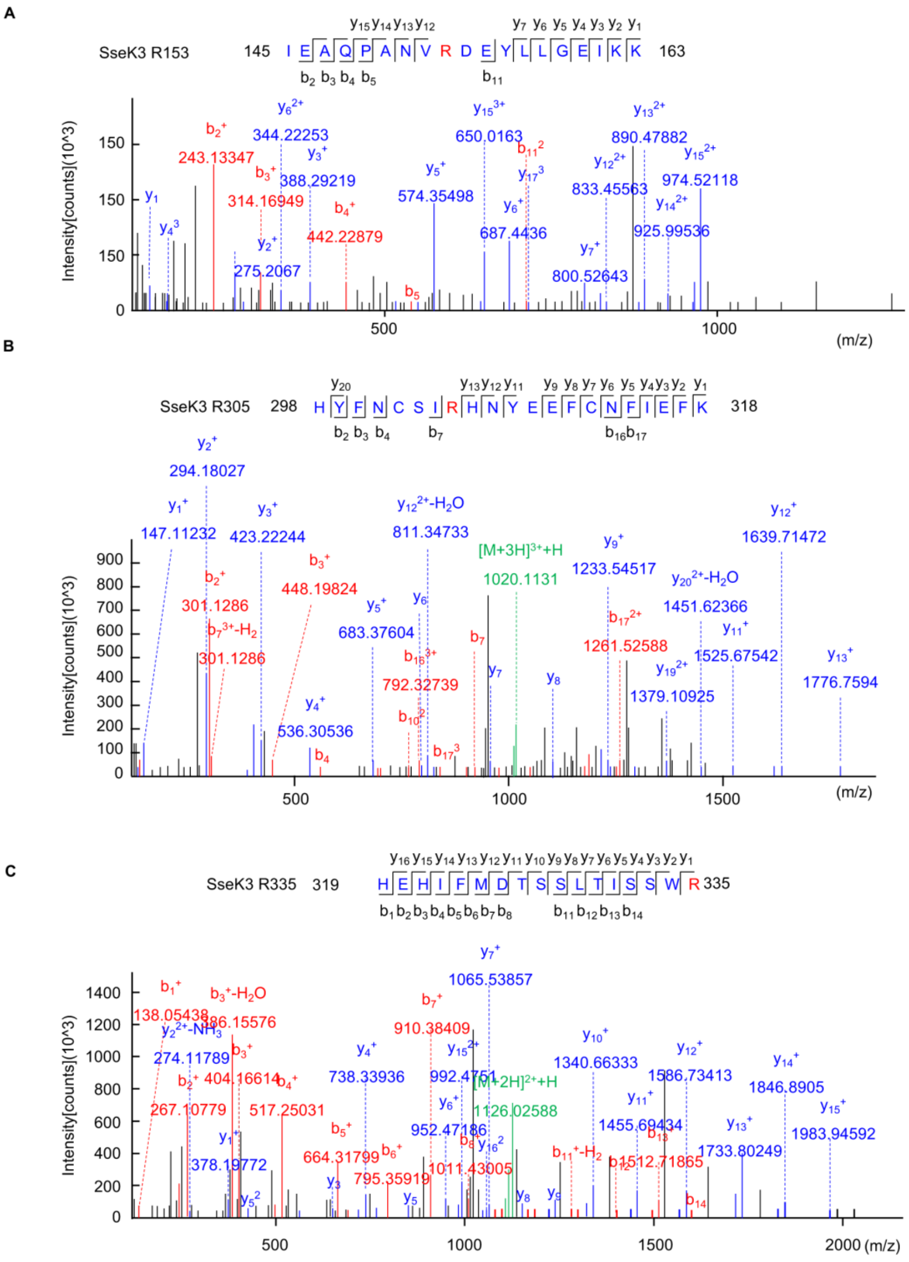

Supplementary Figure 4 HCD analysis of the peptides GlcNAcylated by SseK3. HCD mass spectrum of Arg153 **(A)**, Arg305 **(B)**, and Arg335 **(C)** containing tryptic peptide from SseK3 in bacteria. The fragmentation patterns of the generated *b* and *y* ions were exhibited along the peptide sequence on top of the spectrum.

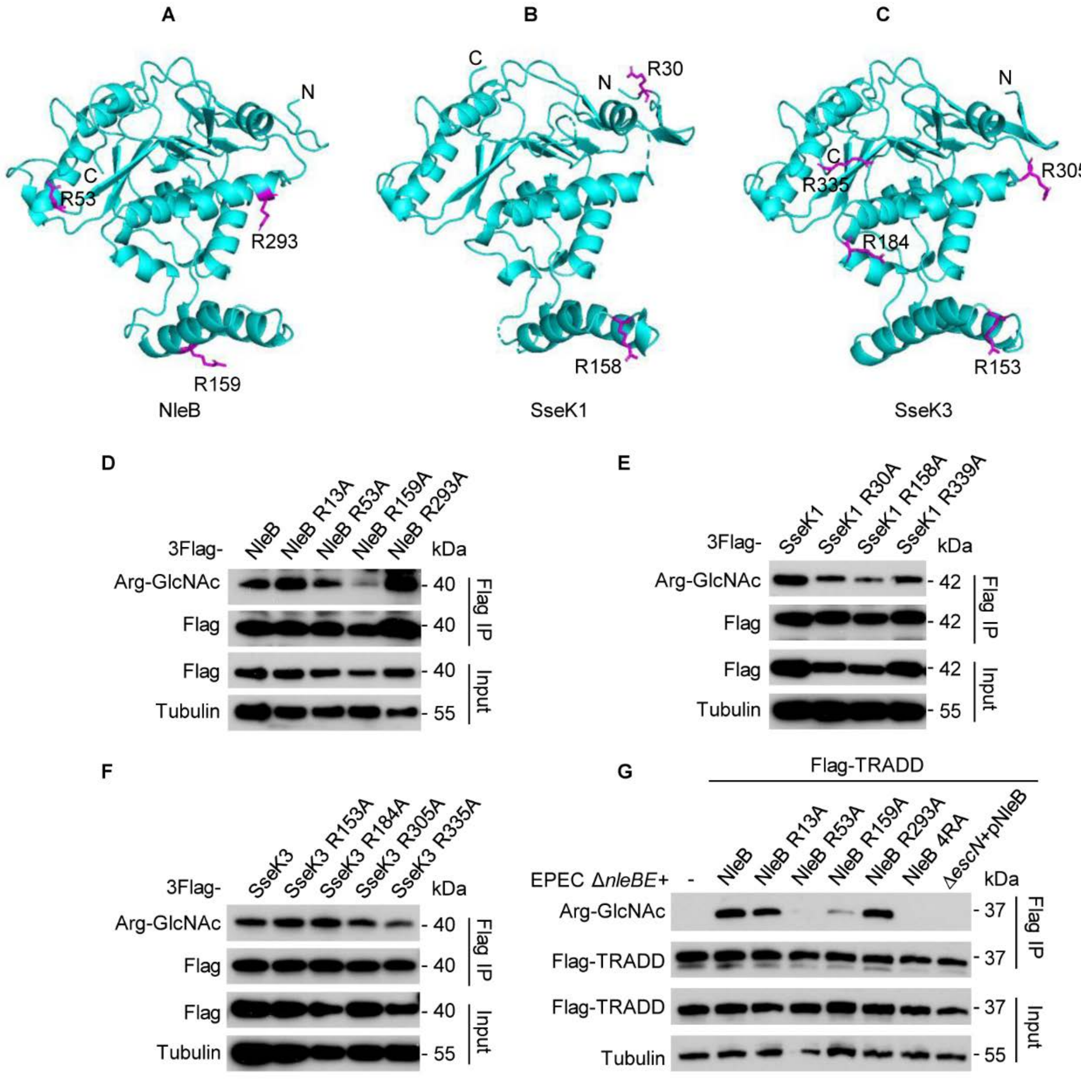

Figure S5 GlcNAcylation activity of single mutations of the auto modification sites of NleB and SseK1/3. (A-C) Overall structure of NleB and SseK1/3. Shown are the cartoon representation of NleB **(A)** (PDB code, 6ACI), SseK1 **(B)** (PDB code, 6IXK), and SseK3 **(C)** (PDB code, 6EYT) in cyan color, respectively. Arginine residues on the auto modification sites are shown as sticks in magenta color. The R13 in NleB and R339 in SseK1 are not shown because they are not visible in the whole structure. **(D-F)** GlcNAcylation activity of single mutations of the auto modification sites of NleB and SseK1/3. 293T cells were transfected with the indicated plasmids. Immunoprecipitants (Flag IP) and cell lysates (Input) were loaded onto SDS-PAGE gels, followed by immunoblot with anti-Flag, anti-Arg-GlcNAc antibody, and a loading control anti-tubulin. **(G)** Modification of protein substrates by NleB and its single mutation during bacterial infection. 293T cells transfected with indicated plasmids were infected with EPEC strains and subjected to anti-Flag immunoprecipitation and immunoblotting. Data in (D-G) are representative from at least three repetitions.

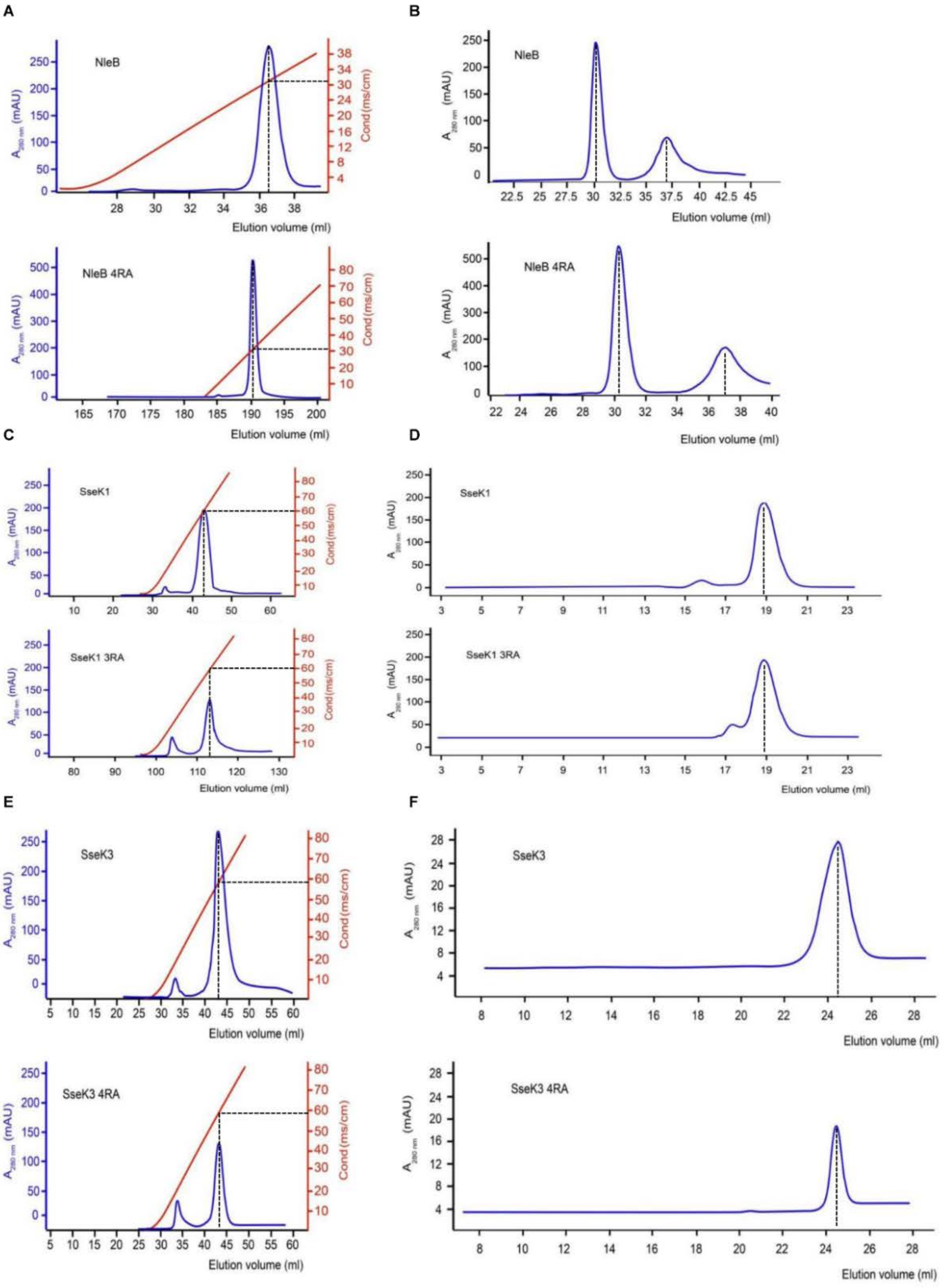

Supplementary Figure 6 Biochemical characterization of NleB/SseK and their RA mutants. (A, B) Ion-exchange **(A)** and gel-filtration **(B)** chromatography of recombinant wild type NleB or an indicated NleB 4RA mutant protein. **(C, D)** Ion-exchange **(C)** and gel-filtration **(D)** chromatography of recombinant wild type SseK1 or an indicated SseK1 3RA mutant protein. (**E, F)** Ion-exchange **(E)** and gel-filtration **(F)** chromatography of recombinant wild type SseK3 or an indicated SseK3 4RA mutant protein.

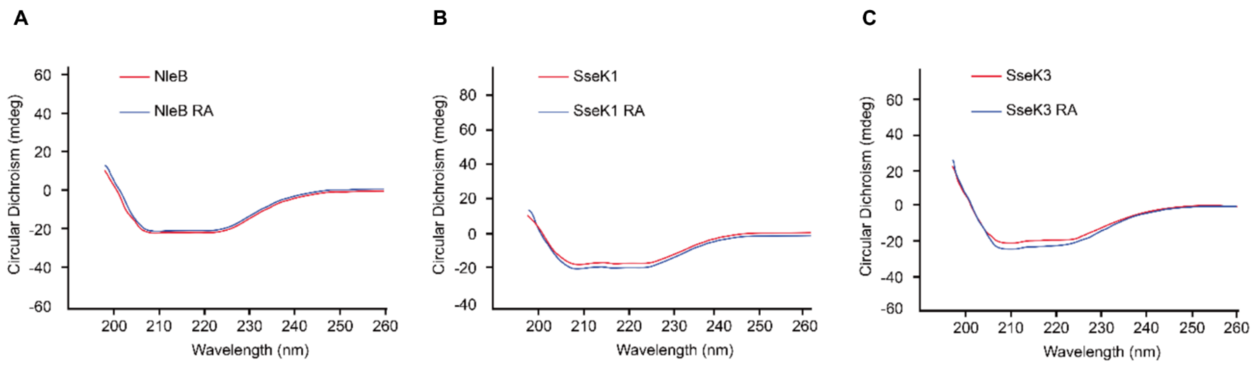

Supplementary Figure 7 Circular dichroism (CD) spectra of NleB/NleB RA (A), SseK1/SseK1 RA (B), and SseK3/SseK3 RA (C). All proteins were prepared at a concentration of 0.22 mg/ml in 10 mM sodium phosphate pH 7.4.

Table S1 EPEC strains used in this study

Name	Reference
E2348/69	(Li et al., 2013)
EPEC Δ*escN*	(Li et al., 2013)
SC3909	(Li et al., 2013)
SC3909+pNleB	(Li et al., 2013)
SC3909+pNleB-DxD	(Li et al., 2013)
SC3909+pNleB-Flag	This study
SC3909+pNleB-DxD-Flag	This study
SC3909+pNleB-HA	This study
SC3909+pNleB-DxD-HA	This study
Δ*escN*+pNleB-HA	This study
Δ*escN*+pNleB-Flag	This study
SC3909+pNleB (4RA)-HA	This study
SC3909+pNleB R13A-HA	This study
SC3909+pNleB R53A-HA	This study
SC3909+pNleB R159A-HA	This study
SC3909+pNleB R293A-HA	This study

Table S2 *Salmonella* strains used in this study

Name	Reference
Salmonella enterica serovar Typhimurium SL1344	This study
SL1344+Vector	This study
SL1344 Δ*sseK1/2/3*+Vector	This study
SL1344 Δ*sseK1/2/3*+pSseK1-Flag	This study
SL1344 Δ*sseK1/2/3*+pSseK1 (3RA)-Flag	This study
SL1344 Δ*sseK1/2/3*+pSseK2-Flag	This study
SL1344 Δ*sseK1/2/3*+pSseK3-Flag	This study
SL1344 Δ*sseK1/2/3*+pSseK3 (4RA)-Flag	This study
SL1344 Δ*sseK1/2/3*+pSseK3-DxD-Flag	This study

Table S3 Plasmids used in this study

Plasmid	Reference
pET28a-SseK1-Flag	This study
pET28a-SseK2-Flag	This study
pET28a-SseK3-Flag	This study
pET28a-SseK3-DxD-Flag	This study
pET28a-SseK1 (3RA)-Flag	This study
pET28a-SseK3 (4RA)-Flag	This study
pTRC99A	This study
pTRC99A-NleB	This study
pTRC99A-NleB-DxD	This study
pTRC99A-NleB-DxD-Flag	This study
pTRC99A-NleB-HA	This study
pTRC99A-NleB-DxD-HA	This study
pTRC99A-NleB (4RA)-HA	This study
pTRC99A-NleB R13A-HA	This study
pTRC99A-NleB R53A-HA	This study
pTRC99A-NleB R159A-HA	This study
pTRC99A-NleB R293A-HA	This study
pCS2-1Flag-TRADD	(Li et al., 2013; Ding et al., 2019)

pCS2-1Flag-TRADD (95-312)	(Li et al., 2013; Ding et al., 2019)
pCS2-1Flag-FADD	(Li et al., 2013)
pCS2-1Flag-RIPK1 DD	(Li et al., 2013)
pCS2-1Flag-TNFR1 (350-445)	(Li et al., 2013)
pCS2-3Flag-TRADD	(Li et al., 2013)
pCS2-3Flag-TRADD (95-312)	(Li et al., 2013)
pCS2-3Flag-NleB	This study
pCS2-3Flag-NleB (4RA)	This study
pCS2-3Flag-NleB R13A	This study
pCS2-3Flag-NleB R53A	This study
pCS2-3Flag-NleB R159A	This study
pCS2-3Flag-NleB R293A	This study
pCS2-3Flag-SseK1	This study
pCS2-3Flag-SseK1 (3RA)	This study
pCS2-3Flag-SseK1 R30A	This study
pCS2-3Flag-SseK1 R158A	This study
pCS2-3Flag-SseK1 R339A	This study
pCS2-3Flag-SseK2	This study
pCS2-3Flag-SseK3	This study
pCS2-3Flag-SseK3 (4RA)	This study

pCS2-3Flag-SseK3 R153A	This study
pCS2-3Flag-SseK3 R184A	This study
pCS2-3Flag-SseK3 R305A	This study
pCS2-3Flag-SseK3 R335A	This study
pCS2-EGFP-NleB	(Li et al., 2013; Ding et al., 2019)
pCS2-EGFP-NleB-DxD	(Li et al., 2013; Ding et al., 2019)
pCS2-EGFP-SseK1	(Li et al., 2013; Ding et al., 2019)
pCS2-EGFP-SseK2	This study
pCS2-EGFP-SseK3	This study
pET28a-His	This study
pET28a-His-NleB	This study
pET28a-His-NleB (4RA)	This study
pET28a-His-SseK1	This study
pET28a-His-SseK1 (3RA)	This study
pET28a-His-SseK2	This study
pET28a-His-SseK3	This study
pET28a-His-SseK3 (4RA)	This study

Ding, J., Pan, X., Du, L., Yao, Q., Xue, J., Yao, H., et al. (2019). Structural and Functional Insights into Host Death Domains Inactivation by the Bacterial Arginine GlcNAcyltransferase Effector. *Mol Cell* 74(5)**,** 922-935 e926. doi: 10.1016/j.molcel.2019.03.028.

Li, S., Zhang, L., Yao, Q., Li, L., Dong, N., Rong, J., et al. (2013). Pathogen blocks host death receptor signalling by arginine GlcNAcylation of death domains. *Nature* 501(7466)**,** 242-246. doi: 10.1038/nature12436.
